# Bacteria Contaminants Detected by Organic Inverter-Based Biosensors

**DOI:** 10.3390/polym16111462

**Published:** 2024-05-22

**Authors:** Po-Hsiang Fang, Han-Chun Chang, Horng-Long Cheng, Chih-Chia Huang, Shuying Wang, Ching-Hao Teng, Zi-Chun Chia, Hai-Pang Chiang, Jrjeng Ruan, Wei-An Shih, Wei-Yang Chou

**Affiliations:** 1 Department of Photonics, National Cheng Kung University, Tainan 70101, Taiwan; 2 Department of Microbiology and Immunology, Institute of Basic Medical Sciences, College of Medicine, National Cheng Kung University, Tainan 70101, Taiwan; 3 Institute of Molecular Medicine, National Cheng Kung University, Tainan 70101, Taiwan; 4 Department of Optoelectronics and Materials Technology, National Taiwan Ocean University, Keelung 20224, Taiwan; 5 Department of Materials Science and Engineering, National Cheng Kung University, Tainan 70101, Taiwan

**Keywords:** polymer, sensors, organic complementary inverter, bacteria, sensitive detection, rapid detection

## Abstract

The importance of bacteria detection lies in its role in enabling early intervention, disease prevention, environmental protection, and effective treatment strategies. Advancements in technology continually enhance the speed, accuracy, and sensitivity of detection methods, aiding in addressing these critical issues. This study first reports the fabrication of an inverter constructed using crosslinked-poly(4-vinylphenol) (C-PVP) as the dielectric layer and an organic complementary metal-oxide semiconductor (O-CMOS) based on pentacene and N,N′-ditridecylperylene-3,4,9,10-tetracarboxylic diimide (PTCDI-C_13_) as a diagnostic biosensor to rapidly detect bacterial concentration. Bacteria including *Escherichia coli* O157, *Staphylococcus aureus* ATCC25922, and *Enterococcus faecalis* SH-1051210 were analysed on the inverters at an ultra-low operating voltage of 2 V. The high density of negative charge on bacteria surfaces strongly modulates the accumulated negative carriers within the inverter channel, resulting in a shift of the switching voltage. The inverter-based bacteria sensor exhibits a linear-like response to bacteria concentrations ranging from 10^2^ to 10^8^ CFU/mL, with a sensitivity above 60%. Compared to other bacterial detectors, the advantage of using an inverter lies in its ability to directly read the switching voltage without requiring an external computing device. This facilitates rapid and accurate bacterial concentration measurement, offering significant ease of use and potential for mass production.

## 1. Introduction

Bacterial contamination is a global health and environmental problem that not only has negative impacts on human sustainable development, but also has serious consequences for animals, the environment, economy, and society [1,2,3]. Pathogenic bacteria are prevalent and widely distributed in the environment, including in soil, water, air, food, and various animals and human bodies. These serve as potential sources of bacterial infections, with most bacterial infections being transmitted through contaminated water, food, or infected animals or humans [4]. Bacteria can be classified into two major categories based on their cell wall structure and composition: Gram-negative bacteria and Gram-positive bacteria. These two types of bacteria exhibit significant differences in their cell wall structure, which play a crucial role in maintaining cellular integrity and withstanding external environments. The cell wall of Gram-negative bacteria consists of two lipid membrane layers, including the inner cytoplasmic membrane and the outer membrane. The cytoplasmic membrane is a typical bilayer of lipid molecules, while the outer membrane is composed of a lipid bilayer with embedded lipopolysaccharide (LPS) molecules. LPS molecules possess both hydrophilic and hydrophobic properties, enabling them to stabilize the outer membrane structure and provide the cell wall with excellent stability and barrier function, effectively protecting the bacteria from antibiotics and other exogenous substances. Additionally, the phosphate groups and carboxyl groups in the LPS molecules confer a negative charge to the bacterial cell surface. Between the two lipid membrane layers lies a thin peptidoglycan layer, which also contributes to the cell wall structure. In contrast, the cell wall structure of Gram-positive bacteria is predominantly composed of a thick peptidoglycan layer, underneath which lies the cytoplasmic membrane. The peptidoglycan layer is tightly composed of cross-linked glycoproteins and peptide chains, making the cell wall extremely rigid and robust. Furthermore, the cell wall also contains polymerized hydrophilic teichoic acid molecules, whose phosphate groups impart a negative charge to the cell surface [5]. The most common bacterial pathogens include *Escherichia coli* O157:H7, *Staphylococcus aureus*, *Campylobacter*, *Listeria monocytogenes*, *Salmonella*, *Bacillus anthracis*, *Bacillus cereus*, *Vibrio cholera*, *Clostridium*, and others [6]. Controlling bacterial contamination requires substantial resources and manpower. If bacterial contamination is not handled properly, it can indirectly affect a country’s international image and reputation, which could impact international exchange and trade, ultimately affecting the country’s economy. In general, bacterial contamination has become a global concern, and it is necessary to further explore and develop effective methods to monitor and control bacterial contamination to ensure public health and environmental safety. However, there are challenges and limitations to detecting and monitoring bacterial contamination. Traditional methods such as bacterial culture and polymerase chain reaction (PCR) have limitations. The bacterial culture is the most time-consuming process, often taking several days to obtain results, while PCR technology may produce false positive results, affecting the accuracy of the test [7,8,9,10]. Additionally, environmental or microbial samples require complex sample preparation steps such as concentration and purification, which may lead to sample distortion and contamination. The issues of false results and low sensitivity restrict their application in bacterial detection. Sensitivity is a crucial parameter as a single bacterium can be the cause of infection. Failure in pathogen detection increases the risk of bacterial transmission, resulting in fatalities and illnesses [11,12]. In certain situations where samples are limited, it is necessary to develop faster, more sensitive, and more specific detection methods to effectively monitor and control bacterial contamination.

Biosensors, a burgeoning branch of sensor technology, seamlessly blend biological techniques with electronic detection. Their operational framework involves harnessing biological components to identify or detect target molecules, coupled with the generation of detectable signals through physical and chemical elements. In essence, this technology transforms biological reactions into precisely measurable signals, making it a highly sophisticated analytical device. The foundational structure of biosensors comprises biological sensing elements and sensors responsible for identifying target samples and translating biological responses into readable signals [13]. Among the various approaches, electrochemical biosensors stand out as an advanced method for detecting and estimating bacterial pathogens, representing one of the most researched techniques in biosensor development. These biosensors rely on changes in electrical properties, such as current, potential, impedance, and conductivity, resulting from interactions between biological receptors and analytes [14,15,16]. However, electrochemical biosensors face challenges related to low reproducibility and stability [17]. In recent years, with the development of science and technology, organic semiconductors have been widely used in various organic electronic devices, such as organic light-emitting diodes (OLEDs) [18,19], organic solar cells [20,21], and organic field-effect transistors (OFETs) [22,23]. OLEDs are widely used in display and lighting applications, while OFETs can be applied in the field of biological detection. OFETs operate based on a three-terminal structure, controlling the flow of charge carriers through organic semiconductor materials by applying an external electric field [24]. The fundamental principle of OFET biosensors involves modulating the electrical characteristics of the semiconductor based on the attachment of biological analytes to the transistor’s surface. This modulation leads to changes in the transistor’s electrical output, allowing for measurement and correlation with the concentration of biological analytes present in the sample. OFET biosensors have been used to detect various biological analytes, including proteins, DNA, and cells [25]. Compared with traditional inorganic semiconductor transistors, OFETs have the advantages of low cost, flexibility, and biocompatibility [26,27,28,29]. In terms of biological detection, OFET’s materials are mostly organic materials, which have higher sensitivity and detection limits and can detect extremely small biological molecules. By modifying the surface of organic semiconductor with biomolecules or biological systems, highly specific detection of target molecules and rapid response to the presence and concentration of target molecules can be achieved [22,30,31,32,33]. While most biosensors currently use graphene transistors to differentiate bacteria based on saturation current changes, there has been limited use of organic semiconductors as active layer materials [34,35,36]. In the year 2019, Dye and their research team introduced a bacterium detection method boasting an impressive sensitivity of detecting as low as 10^3^ bacteria per millilitre (CFU/mL). This pioneering approach hinged upon the modulation of the threshold voltage (*V*_th_) in *n*-type OFETs, enabling the differentiation of various bacterial species [37]. However, the change in *V*_th_ of *n*-type OFET with bacterial concentration was not significant and had no functional correlation. Furthermore, the threshold voltage must be fitted from the transfer electrical curve, which increases the error in calculating bacterial concentration and consumes energy and time. The influence of bacteria on *p*-type OFET properties has not been explored in the literature. OFET-based biosensors offer a simple and convenient detection method, eliminating the need for complex sample processing and sophisticated instrument setup. As a result, they possess vast potential for applications in the field of bio-detection.

In this study, we fabricated a low-voltage driven organic inverter, namely complementary metal–oxide–semiconductor (O-CMOS) on flexible polyimide (PI) substrates. This flexibility offers several advantages, including the potential for developing portable and disposable biosensor devices [38,39]. The active layers consisted of *p*-type pentacene and *n*-type N,N′-ditridecylperylene-3,4,9,10-tetracarboxylic diimide (PTCDI-C_13_), which can serve as a bacterial sensor. The addition of crosslinked-poly(4-vinylphenol) (C-PVP) as the insulating layer enhances the stability of the inverter’s performance while reducing the potential impact of bacterial culture liquid when introduced into the device, making it more suitable for rapid and accurate bacterial detection. Various bacteria were introduced into the inverter to analyse their effect on carrier transport under ultra-low voltage operation (2 V). Subsequently, different concentrations of bacteria were introduced into the *n*-type channel of the O-CMOS. The switching voltage offset of the O-CMOS, which can be directly and rapidly read, gradually decreased with decreasing bacterial concentration. Even when the bacterial concentration decreased to approximately 10^2^ CFU/mL, the O-CMOS device reliably detected the presence of bacteria, indicating its extremely high sensitivity for rapid detection. The inverter-based O-CMOS biosensor provides a label-free and low-cost solution suitable for mass production for bacterial detection. In the future, this sensor fabricated on polymer substrate could be further applied to bacterial detection in river water or other liquid environments, facilitating the establishment of an efficient monitoring system and rapid response mechanism to support research and innovation related to sustainable development.

## 2. Materials and Methods

### 2.1. Device Fabrication and Characterization

**Figure 1:**
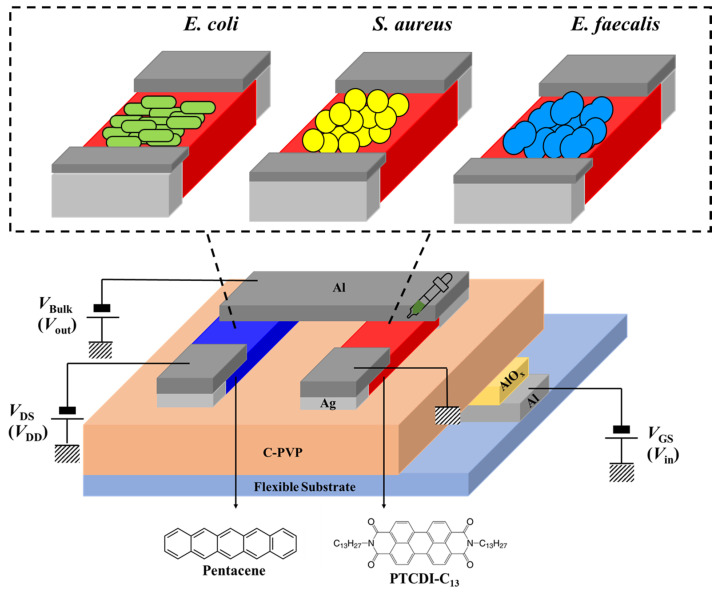
Schematic representation of the fabrication process for a flexible organic inverter-based biosensor.

A bacterial sensor was fabricated using ultra-low-voltage-operated organic inverters with PTCDI-C_13_ as *n*-type active materials and pentacene as *p*-type active materials at a bottom-gate top-contact configuration on a 60 µm-thick PI substrate (2 cm × 2 cm). Its gate dielectric was constructed by bi-layer (Al_2_O_3_/C-PVP) dielectric systems. After cleaning the PI substrate, an 80 nm thick Al film was thermally deposited on the substrate acting as a gate electrode patterned by a shadow mask. The Al gate electrode was treated with oxygen plasma at 200 W for 10 s to form a thin Al_2_O_3_ layer with a high dielectric constant. A blend of poly(4-vinylphenol) (PVP) and poly(melamine-co-formaldehyde) (PMF) with a weight ratio of 12:4 was dissolved in 1-butanol at a concentration of 1 wt.%, which was spun coated on the Al_2_O_3_, and baked at 180 °C for 4 h to form a C-PVP thin film. Both Al_2_O_3_ and C-PVP acted as the dielectric layer. PTCDI-C_13_ and pentacene films (60 nm) were deposited on the C-PVP thin film as the *n*-type and *p*-type active layers, respectively. After depositing the active layers, the deposition of an 80 nm-thick Al/Ag composite film was used as the source and drain electrode on the active layers through a shadow mask with a channel length (*L*) and width (*W*) of 100 and 3000 μm for *p*-type OFETs and 100 and 1000 μm for *n*-type OFETs, respectively. The structures of the OFETs and organic inverter used in this study are shown in Figure 1.

The electrical characteristics of the OFETs and O-CMOS were measured by a Keithley 4200 semiconductor characterization system (Keithley Instruments, Cleveland, OH, USA) inside a N_2_-filled glove box. The surface morphologies of the thin films were detected by atomic force microscopy (AFM, Park XE-100, Park Systems, Suwon, Republic of Korea).

### 2.2. Bacterial Cultivation and Preparation

Two different types of Gram-positive bacteria, namely, *Enterococcus faecalis* (*E. faecalis*) and *Staphylococcus aureus* (*S. aureus*), and one Gram-negative bacterial species, namely *Escherichia coli* (*E. coli*), were used for bacterial sensing considered. All bacteria were grown in a Luria–Bertani (LB) medium at 37 °C and shaken at 200 rpm for 24 h. The bacterial solution was then passed through a centrifuge at 8500 rpm for 5 min to precipitate the bacteria, and the bacterial pellet was then aspirated and dissolved in 1000 μL of deionized (DI) water to prepare stock bacterial solution. The bacterial solution was first measured for optical density at 600 nm to estimate bacterial number, and then validated by spreading 100 μL of each sample on individual agar plates and incubated at 37 °C overnight to obtain the actual CFU of each bacterial solution. Then, the bacterial solutions were serially diluted (10^2^, 10^4^, and 10^8^ CFU/mL) in DI water for a bacterial sensing test by OFETs and O-CMOS sensors.

## 3. Results and Discussion

### 3.1. Surface Morphology

**Figure 2:**
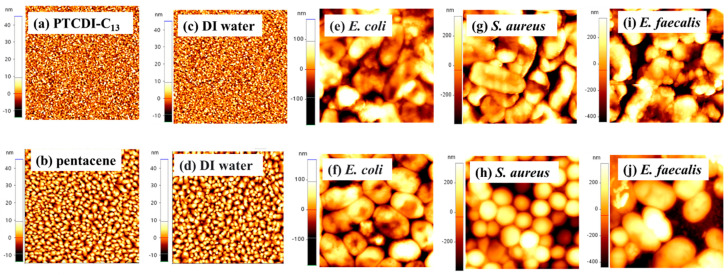
The AFM images of PTCDI-C_13_ (upper panel) and pentacene (lower panel) films under the conditions of (**a**,**b**) native surfaces, (**c**,**d**) dripping DI water, (**e**,**f**) dripping *E. coli*, (**g**,**h**) dripping *S. aureus*, and (**i**,**j**) dripping *E. faecalis*, respectively. The scan is 5 μm × 5 μm in size.

DI water, *E. coli*, *S. aureus*, and *E. faecalis* (bacterial concentration is 10^8^ CFU/mL) were dropped on the surfaces of PTCDI-C_13_ and pentacene films, and their surface morphologies were recorded by AFM. Figure 2a,b show the surface morphologies of PTCDI-C_13_ and pentacene films grown on the C-PVP dielectric layer. The grain size of the PTCDI-C_13_ film was smaller and denser than that of the pentacene film. Some pin holes were observed on the surface of the pentacene film. When the DI water was dropped on the PTCDI-C_13_ and pentacene films, the surface morphologies of the two films did not significantly change from the original PTCDI-C_13_ and pentacene films in the AFM images (Figure 2c,d). Although the surface morphology of the pentacene film dropped with water did not change, the saturation currents of the pentacene- and PTCDI-C_13_-based OFETs slightly decreased after they were dripped with water (Figure S1). As shown in Figure 2e–j, when we dropped the bacteria onto the organic semiconductor films, bacteria only survived on the surface of the semiconductor films because micron-sized bacteria cannot penetrate the film to alter the morphology. The surface morphologies of *E. coli* and *E. faecalis* on the pentacene films exhibited a typical rod shape (Figure 2f,j), while that of *S. aureus* had a normal circle shape (Figure 2h). However, when the three bacteria were dropped on the surfaces of PTCDI-C_13_ films, the cell morphologies differed from their typical morphology. The difference in surface tension between pentacene and PTCDI-C_13_ could change the surface morphology.

### 3.2. Electrical Measurements of OFETs

**Figure 3:**
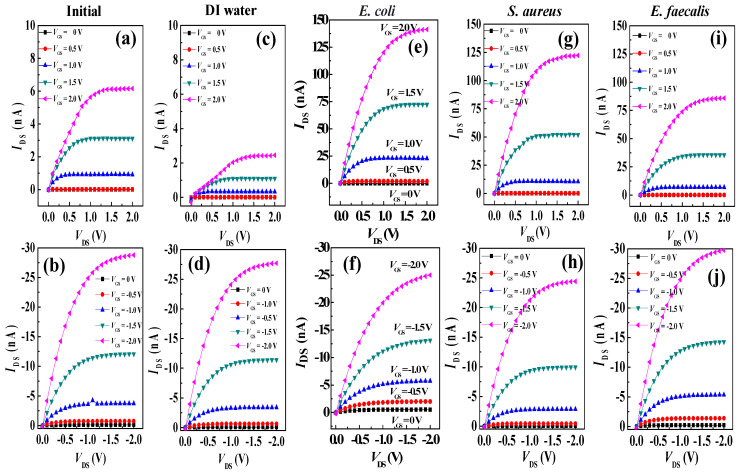
The output characteristics of PTCDI-C_13_- (upper panel) and pentacene (lower panel)-based OFET biosensors to the addition of bacteria, where (**a**,**b**) are non-dropping bacteria, (**c**,**d**) are dropping DI water, (**e**,**f**) are dropping *E. coli*, (**g**,**h**) are dropping *S. aureus*, and (**i**,**j**) are dropping *E. faecalis*.

Figure 3 and Figure S2 show the comparisons of the electrical characteristics of PTCDI-C_13_- and pentacene OFETs before and after dropping DI water or different bacteria within the channel, respectively. When the PTCDI-C_13_-based device was dropped with DI water, the subthreshold swing (*S.S.*) became worse compared with the original device and the saturation currents of the transfer curve and output curve were reduced by half (Figure 3a,c and Figure S2a,c). The primary charge carriers in *n*-type organic semiconductors are electrons, which, due to their smaller mass, are more susceptible to being captured by water or oxygen molecules in the environment. When the active layer of the organic semiconductor materials comes into contact with moisture and oxygen, oxidation occurs, generating defects and traps at grain boundaries or interfaces that capture a significant number of electrons. This reduction in free electrons subsequently decreases the conductivity and current transport capability of *n*-type organic semiconductors, resulting in degraded electrical performance [40,41,42]. Therefore, when DI water is applied to the channel of a PTCDI-C_13_ device, the presence of water molecules initiates the aforementioned process, leading to a poorer electrical performance of the device compared to other devices.

When the PTCDI-C_13_-based device was dropped with different bacteria (the number of bacteria was 10^8^ CFU/mL), the transfer curve had a clockwise hysteresis phenomenon due to water infiltration between the dielectric and semiconducting layers, causing it to generate a trap mode. When the PTCDI-C_13_ device was dropped with different bacteria, the saturation current of the transfer curve was greatly increased compared with the electrical characteristics of the original device. The device dropped with *E. coli* had the highest increase in saturation current, from 1.95 × 10^−8^ A to 4.05 × 10^−7^ A, followed by the device dropped with *S. aureus* (3.51 × 10^−7^ A). Finally, the device dropped with *E. faecalis* had the lowest saturation current of 2.86 × 10^−7^ A (Figure 3e,g,i). Accordingly, the electrical characteristics, including the saturation current, for the *n*-type OFETs after dropping bacteria were an order of magnitude higher than those before dropping bacteria. According to the literature, Gram-negative bacteria carry more negative charges than Gram-positive bacteria [43,44,45,46]. The sensing mechanism of the *n*-type OFETs could be attributed to the conductivity of the *n*-type channel, which is modulated by dropping negatively charged bacteria onto the channel of OFETs. Bacterial cells, such as *Escherichia coli* (*E. coli*), typically possess negatively charged surfaces. In OFETs, the majority carriers in *p*-type semiconductors are holes (positive charges). When bacteria make contact with the surface of a *p*-type semiconductor, the negative charges on the bacterial surfaces attract holes in the semiconductor, leading to hole accumulation at the semiconductor/bacteria interface. Consequently, electrons accumulate on the channel of pentacene-based OFETs, facilitating the recombination of majority carriers, holes, resulting in a slight decrease in the saturation current. As a result, the on/off current ratio of *E. coli*-dropped OFETs was slightly lower than that of DI-dropped OFETs, as presented in Table 1.

In the case of PTCDI-C13-based OFETs, the device with *S. aureus* dropped on it had a higher saturation current than the device with *E. faecalis*, indicating that the negative charge density on the surface of *S. aureus* is higher than that of *E. faecalis*, as displayed in Figure 3g,i. However, dropping bacteria with high negative charge density on the pentacene surface is not conducive to hole accumulation in the channels of *p*-type devices. As shown in Figure 3h,j, the saturation current of the pentacene-based OFET with *S. aureus* is lower than that with *E. faecalis*.

**Figure 4:**
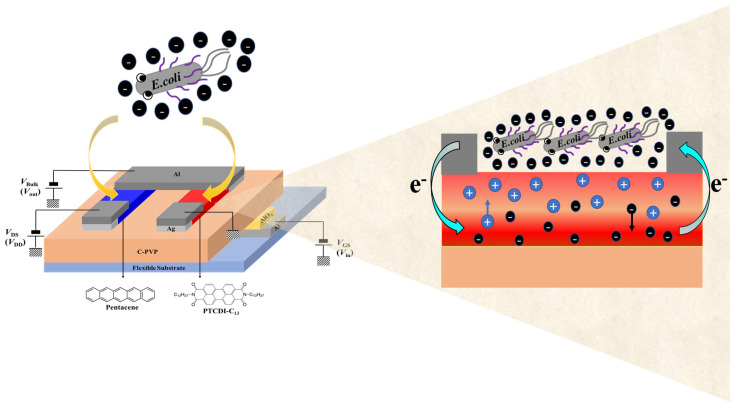
The sensing mechanism of O-CMOS-based biosensors in the presence of bacteria dropped in the channel, taking *E. coli* as an example.

From Figure S2e, when *E. coli* cells were dropped into the PTCDI-C_13_-based devices, the threshold voltage (*V*_th_) of the OFETs tended to normally turn on, that is, a negative *V*_th_; therefore, the highly negatively charged *E. coli* enhanced electron accumulation in the active channel, leading to early turn on of the OFETs. The physical mechanism is shown in Figure 4. The threshold voltage is defined as the minimum gate voltage required to accumulate sufficient carriers in the semiconductor layer and form a channel between the semiconductor and dielectric layers. The *V*_th_ can be obtained using the transistor transfer curve. Plotting *V*_GS_ on the *x*-axis and $\sqrt{I_{DS}}$ on the *y*-axis, and obtaining the intercept using the tangent method, the intercept value represents the *V*_th_ [47]. A subthreshold swing (*S.S.*) is a ratio of change in the gate biasing to the change in the drain current in logarithmic scale, which can be expressed as [48]

(1) $$S.S.={\left[\frac{\partial (\log I_{DS})}{\partial V_{GS}}\right]}^{-1}$$

As the devices were dropped with *S. aureus* and *E. faecalis* cells (having less charge), the *V*_th_ maintained the normal operation mode, that is, positive *V*_th_ (Figure S2g,i). Therefore, Gram-negative and Gram-positive bacteria can be distinguished by the *V*_th_ of the PTCDI-C_13_-based OFETs. When the pentacene-based devices were dropped with DI water or different bacteria, the saturation currents of the transfer and output curves had no significant change and slightly variations were observed for *S.S.* (Figure 3 and Figure S2). The negative charges on the bacterial surface did not contribute to the accumulation of the positive charges in the channel of *p*-type OFET. As such, the electrical properties of the *p*-type OFETs did not improve after dropping bacteria. All electrical characteristic parameters of the two-type OFETs are presented in Table 1.

### 3.3. Determination of Bacteria Concentration

**Figure 5:**
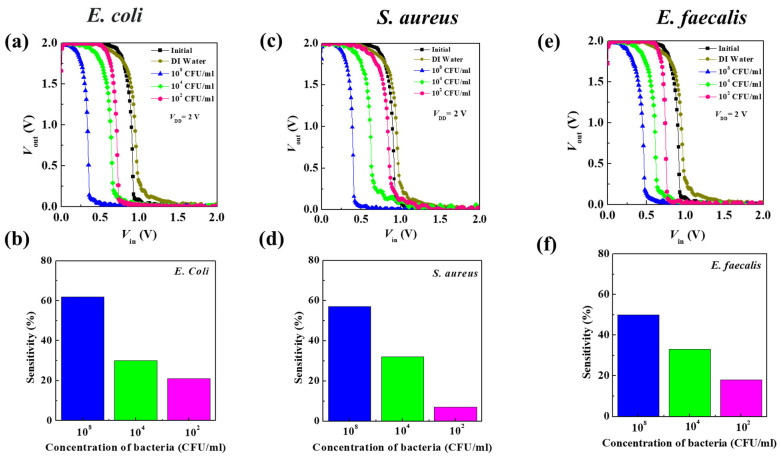
The electrical performance of O-CMOS decreased in different concentrations of bacteria. (**a**) The voltage transfer curves of the devices dripped with *E. coli*, (**b**) the sensitivity at different *E. coli*’s concentrations, (**c**) the voltage transfer curves of the devices dripped with *S. aureus*, (**d**) the sensitivity at different *S. aureus*’s concentrations, (**e**) the voltage transfer curves of devices dripped with *E. faecalis*, and (**f**) the sensitivity at different *E. faecalis* concentrations.

A bacterial sensor was fabricated by integrating the PTCDI-C_13_- and pentacene-based OFETs to form O-CMOS sensors, in which DI water and bacteria of different concentrations (10^8^, 10^4^, and 10^2^ CFU/mL in sequence) were dropped on the channel. Based on the experimental results, the pentacene-based OFETs had no response to the bacteria, so the operation of the O-CMOS during detection was controlled by bacteria-dropped PTCDI-C_13_-based OFETs. Before dropping the bacteria, the drain power voltage *V*_DD_ = 2 V was applied to O-CMOS, which is a high potential relative to the input voltage (*V*_in_, Figure S3). The *p-*type channel was turned on, and the output voltage (*V*_out_) was equal to *V*_DD_ and at a high level (Figure S3). When the *V*_in_ was gradually increased to *V*_DD_/2, the *n-*type channel was also gradually turned on, causing *V*_out_ to quickly drop to the ground state, that is, the low level. The *V*_out_ of the O-CMOS switched from high-level to low-level voltage and was regarded as the switching voltage (*V*_S_), which is about *V*_DD_/2. The characteristics of the conversion curve and the offset of switching voltage (Δ*V*_S_) were compared among the O-CMOS devices dropped with different concentrations of bacteria (Table 2). At the beginning, the *V*_S_ of O-CMOS was around 0.9 V. When the highest bacterial concentration of *E. coli* of 10^8^ CFU/mL was dropped to the O-CMOS, the *V*_S_ shifted to 0.34 V and the Δ*V*_S_ was 0.56 V (Figure 5a). As shown in Figure 4, the sensing mechanism in the O-CMOS can be attributed to the conductivity of the PTCDI-C_13_ channel being modulated upon coming in contact with negatively charged *E. coli* cells. That is to say, *E. coli,* with a high negative charge, induces a large amount of charge to accumulate on the channel of PTCDI-C_13_, causing the *n*-channel to turn on early [49]. As a result, the *V*_S_ deviates from *V*_DD_/2 and moves toward a lower voltage. As the concentration of *E. coli* decreased, the *V*_S_ gradually shifted toward the *V*_DD_/2 due to the decrease in induced carriers by decreasing the *E. coli* concentration, that is, the linear-like decrease in Δ*V*_S_ with a decreasing *E. coli* concentration. A fast response time is necessary for the detection of bacteria. The short response time is one of the important advantages of the O-CMOS sensor because the switching voltage can be directly read without the need for an external computing device. This enables quick measurement of the bacterial concentration and makes its usage quite convenient. When the sensor was applied to detect *S. aureus* and *E. faecalis*, similar results to the detection of *E. coli* were obtained. The detection of *S. aureus* and *E. faecalis* under the concentration of 10^8^ CFU/mL had a *V*_S_ of 0.39 and 0.45 V, as well as a Δ*V*_S_ of 0.51 and 0.45 V, respectively (Figure 5c,e). The sensitivity is generally used to benchmark the performance of a biosensor and is defined as Equation (2):

(2) $$Sensitivity\;\left(\%\right)=\frac{∆V_{s}}{V_{s_{0}}}\times 100\%$$

where the offset of switching voltage is defined as $∆V_{s}=\left|V_{s}\right.-\left.V_{s0}\right|$, in which *V*_S_ represents the switching voltage value of the O-CMOS bacteria sensor after the bacteria are introduced, and $V_{S_{o}}$ is the value of the switching voltage of the original device [50]. Figure 5b,d,f illustrate the sensitivity of the O-CMOS-based biosensors to *E. coli*, *S. aureus*, and *E. faecalis*. The maximum sensitivity of the O-CMOS-based biosensor for *E. coli* reached 62%, tested by the concentration of 10^8^ CFU/mL. Subsequently, the sensitivity diminished with decreasing bacterial concentrations, notably for *S. aureus*, reaching its minimum at a concentration of 10^2^ CFU/mL. The Δ*V*_S_ had a linear-like relationship to bacterial concentration and the ability to differentiate between Gram-positive and Gram-negative bacteria using sensitivity, which could lead to the rapid detection of bacterial content in water or specific liquid media. Therefore, bacterial content can be estimated from the offset of the O-CMOS switching voltage, achieving the ability of quantitative analysis of biosensors. When the concentration of bacteria was as low as 10^2^ CFU/mL, the Δ*V*_S_ of the O-CMOS sensor was still easily identified to challenge the lowest possible detection limit of 10^2^ CFU/mL, which is important for mass production and practical applications.

## 4. Conclusions

In summary, we integrated PTCDI-C_13_- and pentacene-based OFETs to fabricate O-CMOS label-free biosensors for the detection of *E. coli*, *S. aureus*, and *E. faecalis*. The sensing mechanism relies on the modulation of the conductivity of PTCDI-C_13_ by inducing charge carriers in the channel of O-CMOS after dropping with negatively charged bacteria. According to literature reports, Gram-negative bacteria such as *E. coli* have a higher negative charge on their cell surfaces compared to Gram-positive bacteria such as *S. aureus* and *E. faecalis.* Consequently, this sensor exhibits significantly enhanced electrical performance compared to the pristine device, and the type of bacteria can be distinguished based on the positive or negative shift in the threshold voltage of the PTCDI-C_13_-based OFET. The O-CMOS sensors are designed for detection of bacteria by using the offset of switching voltage, which has a linear-like response to the concentration of bacteria within 10^2^–10^8^ CFU/mL. This study is the first report an O-CMOS based bacteria sensor with a detection limitation of 10^2^ CFU/mL for the detection of bacteria in water. Moreover, the proposed O-CMOS sensor can be fabricated on flexible polyimide substrates and operated at an ultra-low voltage of 2 V. This low-cost and highly sensitive sensor can be easily mass produced. In the future, we will modify the detection capabilities of O-CMOS sensors and OFETs sensors that can be applied to water quality monitoring, pathogenic bacterium detection, and identification of disease-causing bacteria involved in drinking water outbreaks.

## Figures and Tables

**Table 1:** Summary of electrical performances for PTCDI-C_13_- and pentacene-based OFETs.

Organic Semiconductor	Types of Solutions	*V*_th_ (V)	*V*_th_ (V) (Average Value of Five Devices)	On/Off Current Ratio	*S.S.* (V/dec)
PTCDI-C_13_	Non solution	0.38	0.31 ± 0.39	1.37 × 10^4^	0.15
	DI water	0.48	0.50 ± 0.38	1.27 × 10^4^	0.23
	*E. coli*	−0.09	−0.12 ± 0.21	7.85 × 10^6^	0.06
	*S. aureus*	0.06	0.19 ± 0.73	6.45 × 10^5^	0.11
	*E. faecalis*	0.28	0.07 ± 0.28	6.99 × 10^6^	0.08
Pentacene	Non solution	−0.68	−0.56 ± 0.08	1.58 × 10^4^	0.37
	DI water	−0.44	−0.30 ± 0.19	3.23 × 10^3^	0.46
	*E. coli*	−0.31	−0.39 ± 0.17	1.68 × 10^3^	0.69
	*S. aureus*	−0.37	−0.25 ± 0.71	8.51 × 10^3^	0.42
	*E. faecalis*	−0.43	−0.28 ± 0.51	4.01 × 10^3^	0.52

**Table 2:** The switching voltage offset (Δ*V*_S_) of the O-CMOS device after being dropped on different concentrations of bacteria.

Concentration of Bacteria (CFU/mL)	Δ*V*_S_ (V)		
	*E. coli*	*S. aureus*	*E. faecalis*
10^8^	0.56	0.51	0.45
10^4^	0.27	0.29	0.30
10^2^	0.19	0.06	0.16

## Data Availability

Data are contained within the article and Supplementary Materials.

## Supplementary Materials

The following supporting information can be downloaded at: https://www.mdpi.com/article/10.3390/polym16111462/s1, Figure S1. Comparison of electrical properties of (a) PTCDI-C_13_-based and (b) pentacene-based OFETs between native state and after dripping water; Figure S2. The transfer characteristics of PTCDI-C_13_- (upper panel) and pentacene (lower panel) based OFET biosensors to the addition of bacteria, where (a) and (b) non-dropping bacteria, (c) and (d) dropping DI water, (e) and (f) dropping *E. coli*, (g) and (h) dropping *S. aureus*, and (i) and (j) dropping *E. faecalis*; Figure S3. Schematic diagram of O-CMOS operation.

## References

[1] Suthar S., Chhimpa V., Singh S. (2009). Bacterial contamination in drinking water: A case study in rural areas of northern Rajasthan, India. Environ. Monit. Assess..

[2] Korzeniowska A.C., Trawińska B., Tymczyna L., Wencel H.B., Matuszewski Ł. (2021). Microbial contamination of the air in livestock buildings as a threat to human and animal health—A review. Ann. Anim. Sci..

[3] Waldman A.J., Balskus E.P. (2018). The Human Microbiota, Infectious Disease, and Global Health: Challenges and Opportunities. ACS Infect. Dis..

[4] Lacroix J.S., Ricchetti A., Lew D., Delhumeau C., Morabia A., Stalder H., Terrier F., Kaiser L. (2002). Symptoms and clinical and radiological signs predicting the presence of pathogenic bacteria in acute rhinosinusitis. Acta Otolaryngol..

[5] Silhavy T.J., Kahne D., Walker S. (2010). The Bacterial Cell Envelope. Cold Spring Harb. Perspect. Biol..

[6] Tagliaferri T.L., Jansen M., Horz H.-P. (2019). Fighting pathogenic bacteria on two fronts: Phages and antibiotics as combined strategy. Front. Cell. Infect. Microb..

[7] Lim S.H., Mix S., Anikst V., Budvytiene I., Eiden M., Churi Y., Queralto N., Berliner A., Martino R.A., Rhodesa P.A. (2016). Bacterial culture detection and identification in blood agar plates with an optoelectronic nose. Analyst.

[8] Huang X.X., Urosevic N., Inglis T.J.J. (2019). Accelerated bacterial detection in blood culture by enhanced acoustic flow cytometry (AFC) following peptide nucleic acid fluorescence in situ hybridization (PNA-FISH). PLoS ONE.

[9] Higgins J.A., Azad A.F. (1995). Use of polymerase chain reaction to detect bacteria in arthropods: A review. J. Med. Entomol..

[10] Zhang H., Morrison S., Tang Y.W. (2015). Multiplex polymerase chain reaction tests for detection of pathogens associated with gastroenteritis. Clin. Lab. Med..

[11] Law J.W.F., Mutalib N.S.A., Chan K.G., Lee L.H. (2015). Rapid methods for the detection of foodborne bacterial pathogens: Principles, applications, advantages and limitations. Front. Microbiol..

[12] Yadav N., Chhillar A.K., Rana J.S. (2020). Detection of pathogenic bacteria with special emphasis to biosensors integrated with AuNPs. Sens. Int..

[13] Burlage R.S., Tillmann J. (2017). Biosensors of bacterial cells. J. Microbiol. Methods.

[14] Riu J., Giussani B. (2020). Electrochemical biosensors for the detection of pathogenic bacteria in food. Trac. Trends Anal. Chem..

[15] Khan M.Z.H., Hasan M.R., Hossain S.I., Ahommed M.S., Daizy M. (2020). Ultrasensitive detection of pathogenic viruses with electrochemical biosensor: State of the art. Biosens. Bioelectron..

[16] Rani R., Deep A., Mizaikoff B., Singh S. (2020). Copper based organic framework modified electrosensor for selective and sensitive detection of ciprofloxacin. Electroanalysis.

[17] Niu Y., Qin Z., Zhang Y., Chen C., Liu S., Chen H. (2023). Expanding the potential of biosensors: A review on organic field effect transistor (OFET) and organic electrochemical transistor (OECT) biosensors. Mater. Futures.

[18] Kudo K. (2005). Organic light emitting transistors. Curr. Appl. Phys..

[19] Daniso E., Maroh B., Feldbacher S., Mühlbacher I., Schl S., Melpignano P. (2021). Tailoring the chemical functionalization of a transparent polyethylene foil for its application in an OLED-based DNA biosensor. Appl. Surf. Sci..

[20] Xu W., Yang W.K., Guo H.K., Ge L.Y., Tu J.C., Zhen C. (2020). Constructing a TiO_2_/PDA core/shell nanorod array electrode as a highly sensitive and stable photoelectrochemical glucose biosensor. RSC Adv..

[21] Lungenschmied C., Dennler G., Neugebauer H., Sariciftci S.N., Glatthaar M., Meyer T., Meyer A. (2007). Flexible, long-lived, large-area, organic solar cells. Sol. Energy Mater. Sol. Cells.

[22] Tao J.W., Sun W.B., Lu L.H. (2022). Organic small molecule semiconductor materials for OFET-based biosensors. Biosens. Bioelectron..

[23] Hao R.F., Yue Y.F., Li L.L., Ji J.L., Zhang Q., Ding L.F., Sang S.B., Li Q. (2022). P3HT-based organic field effect transistor for low-cost, label-free detection of immunoglobulin G. J. Biotechnol..

[24] Luo L., Liu Z. (2022). Recent progress in organic field-effect transistor-based chem/bio-sensors. View.

[25] Basiricò L., Mattana G., Mas-Torrent M. (2022). Editorial: Organic Electronics: Future Trends in Materials, Fabrication Techniques and Applications. Front. Phys..

[26] Vasimalla S., Subbarao N.V.V., Iyer P.K. (2016). Low voltage, low cost, flexible and balanced ambipolar OFETs based on Br2PTCDI-C18/CuPc fabricated on an Al foil gate substrate with good ambient stability. J. Mater. Chem. C.

[27] Shi W., Guo Y., Liu Y. (2020). When Flexible Organic Field-Effect Transistors Meet Biomimetics: A Prospective View of the Internet of Things. Adv. Mater..

[28] Shen H., Di C.A., Zhu D. (2017). Organic transistor for bioelectronic applications. Sci. China Chem..

[29] Yuvaraja S., Nawaz A., Liu Q., Duba D., Surya S.G., Salama K.N., Sonar P. (2020). Organic field-effect transistor-based flexible sensors. Chem. Soc. Rev..

[30] Li H., Shi Y., Han G., Liu J., Zhang J., Li C., Liu J., Yi Y., Li T., Gao X. (2020). Monolayer Two-dimensional Molecular Crystals for an Ultrasensitive OFET-based Chemical Sensor. Angew. Chem. Int. Ed..

[31] Ohayon D., Inal S. (2020). Organic Bioelectronics: From Functional Materials to Next-Generation Devices and Power Sources. Adv. Mater..

[32] Lin P., Yan F. (2012). Organic Thin-Film Transistors for Chemical and Biological Sensing. Adv. Mater..

[33] Magliulo M., Mallardi A., Gristina R., Ridi F., Sabbatini L., Cioffi N., Palazzo G., Torsi L. (2013). Part per Trillion Label-Free Electronic Bioanalytical Detection. Anal. Chem..

[34] Lin Z., Wu G., Zhao L., Lai K.W.C. (2021). Detection of bacterial metabolic volatile indole using a graphene-based field-effect transistor biosensor. Nanomaterials.

[35] Tan X.B., Yang M.Y., Zhu L., Gunathilaka G., Zhou Z.X., Zhang Y.F., Cheng M.M.C. (2022). Ultrasensitive and Selective Bacteria Sensors Based on Functionalized Graphene Transistors. IEEE Sens. J..

[36] Kim K.H., Park S.J., Park C.S., Seo S.E., Lee J., Kim J.Y., Lee S.H., Lee S.H., Kim J.S., Ryu C.M. (2020). High-performance portable graphene field-effect transistor device for detecting Gram-positive and -negative bacteria. Biosens. Bioelectron..

[37] Dey A., Singh A., Dutta D., Ghosh S.S., Iyer P.K. (2019). Rapid and label-free bacteria detection using a hybrid tri-layer dielectric integrated n-type organic field effect transistor. J. Mater. Chem. A.

[38] Jia D.Y., Jiang L., Cai X.Z., Dong H.L., Meng Q., Tian G.F., Wu D.Z., Li J.Z., Hu W.P. (2013). Large scale, flexible organic transistor arrays and circuits based on polyimide materials. Org. Electron..

[39] Liang Y.Y., Guo T., Zhou L., Offenhäusser A., Mayer D. (2020). Label-Free Split Aptamer Sensor for Femtomolar Detection of Dopamine by Means of Flexible Organic Electrochemical Transistors. Materials.

[40] Griggs S., Marks A., Bristowa H., McCullochab I. (2021). n-Type organic semiconducting polymers: Stability limitations, design considerations and applications. J. Mater. Chem. C.

[41] Hwang D.K., Fuentes-Hernandez C., Fenoll M., Yun M., Park J., Shim J.W., Knauer K.A., Dindar A., Kim H., Kim Y. (2014). Systematic Reliability Study of Top-Gate p- and n-Channel Organic Field-Effect Transistors. ACS Appl. Mater. Interfaces.

[42] Zschieschang U., Amsharov K., Jansen M., Kern K., Klauk H., Weitz R.T. (2015). Separating the impact of oxygen and water on the long-term stability of n-channel perylene diimide thin-film transistors. Org. Electron..

[43] Sonohara R., Muramatsu N., Ohshima H., Kondo T. (1995). Difference in surface properties between Escherichia coli and Staphylococcus aureus as revealed by electrophoretic mobility measurements. Biophys. Chem..

[44] Arakha M., Saleem M., Mallick B.C., Jha S. (2015). The effects of interfacial potential on antimicrobial propensity of ZnO nanoparticle. Sci. Rep..

[45] Wilhelm M.J., Gh M.S., Wu T., Li Y., Chang C.M., Ma J.Q., Dai H.L. (2021). Determination of bacterial surface charge density via saturation of adsorbed ions. Biophys. J..

[46] Feng Z.V., Gunsolus I.L., Qiu T.A., Hurley K.R., Nyberg L.H., Frew H., Johnson K.P., Vartanian A.M., Jacob L.M., Lohse S.E. (2015). Impacts of gold nanoparticle charge and ligand type on surface binding and toxicity to Gram-negative and Gram-positive bacteria. Chem. Sci..

[47] Ortiz-Conde A., Garcı F.J., Liou J.J., Cerdeira A., Estrada M., Yue Y. (2002). A review of recent MOSFET threshold voltage extraction methods. Microelectron. Reliab..

[48] Park J.S., Maeng W.J., Kim H.S., Park J.S. (2012). Review of recent developments in amorphous oxide semiconductor thin-film transistor devices. Thin Solid Films.

[49] Chou W.Y., Peng S.K., Wu F.C., Sheu H.S., Wang Y.F., Huang P.K., Cheng H.L. (2020). Memory characteristics of organic field-effect memory transistors modulated by nano-p–n junctions. J. Mater. Chem. C.

[50] Fang P.H., Wu F.C., Sheu H.S., Lai J.H., Cheng H.L., Chou W.Y. (2021). Analysis of ultrathin organic inverters by using in situ grazing incidence X-ray diffraction under high bending times and low voltage. Org. Electron..

