# Supplementary material for: Bacteria Contaminants Detected by Organic Inverter-Based Biosensors
Source: Polymers (Basel). 2024 May 22;16(11):1462. doi: 10.3390/polym16111462 (PMC11174487; doi:10.3390/polym16111462)
Supplement: Supplementary file 1 [file polymers-16-01462-s001.zip › polymers-2981668-supplementary.pdf]

Bacteria contaminants detected by organic inverter-based biosensors

Po-Hsiang Fang[1], Han-Chun Chang[1], Horng-Long Cheng[1], Chih-Chia Huang[1], Shuying Wang[2], Ching-Hao Teng[3], Zi-Chun Chia[1], Hai-Pang Chiang[4], Jrjeng Ruan[5], Wei-An Shih[3] and Wei-Yang Chou [1,*]

[1]Department of Photonics, National Cheng Kung University, Tainan 70101, Taiwan.
[2]Department of Microbiology and Immunology, Institute of Basic Medical Sciences, College of Medicine, National Cheng Kung University, Tainan 70101, Taiwan.
[3]Institute of Molecular Medicine, National Cheng Kung University, Tainan 70101, Taiwan.
[4]Department of Optoelectronics and Materials Technology, National Taiwan Ocean University, Keelung 20224, Taiwan.
[5]Department of Materials Science and Engineering, National Cheng Kung University, Tainan 701, Taiwan

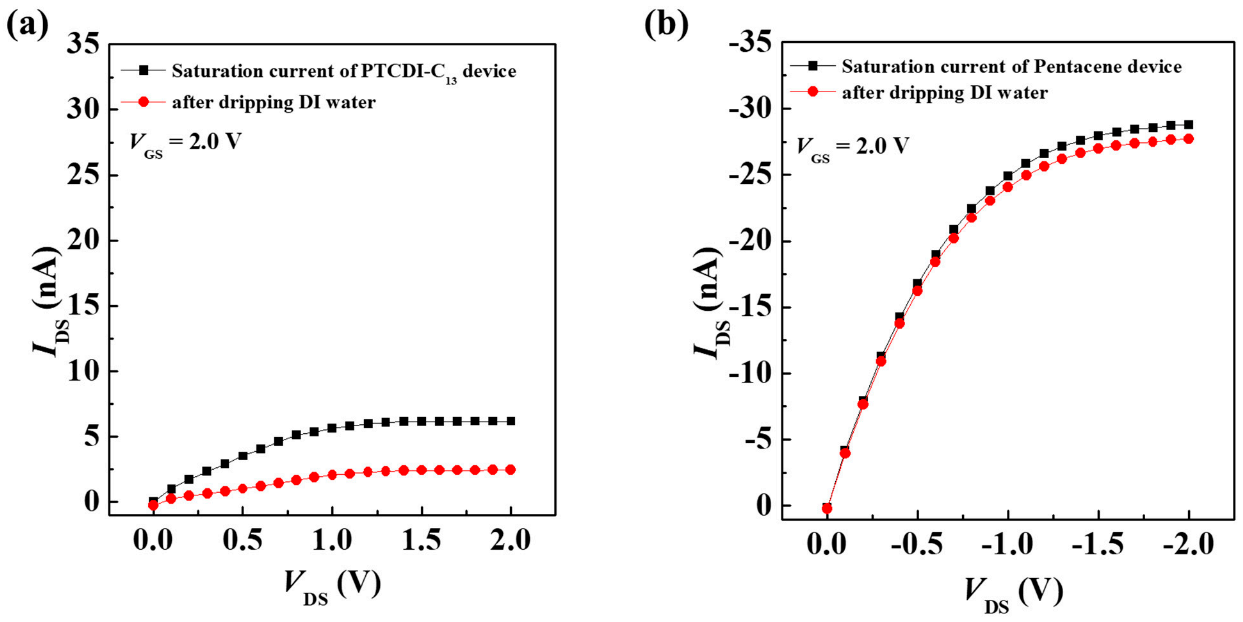

Figure S1. Comparison of electrical properties of (a) PTCDI-C_{13}-based and (b) pentacene-based OFETs between native state and after dripping water.

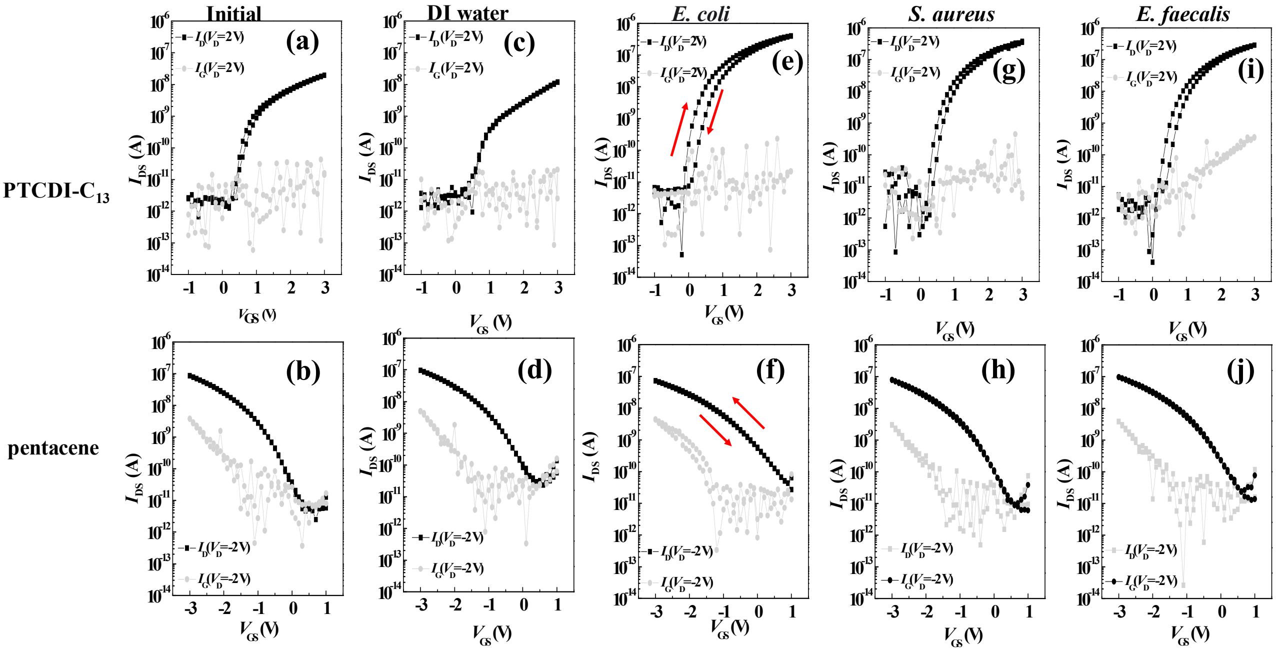

Figure S2. The transfer characteristics of PTCDI-C13- (upper panel) and pentacene (lower panel) based OFET biosensors to the addition of bacteria, where (a) and (b) non-dropping bacteria, (c) and (d) dropping DI water, (e) and (f) dropping *E. coli*, (g) and (h) dropping *S. aureus*, and (i) and (j) dropping *E. faecalis*.

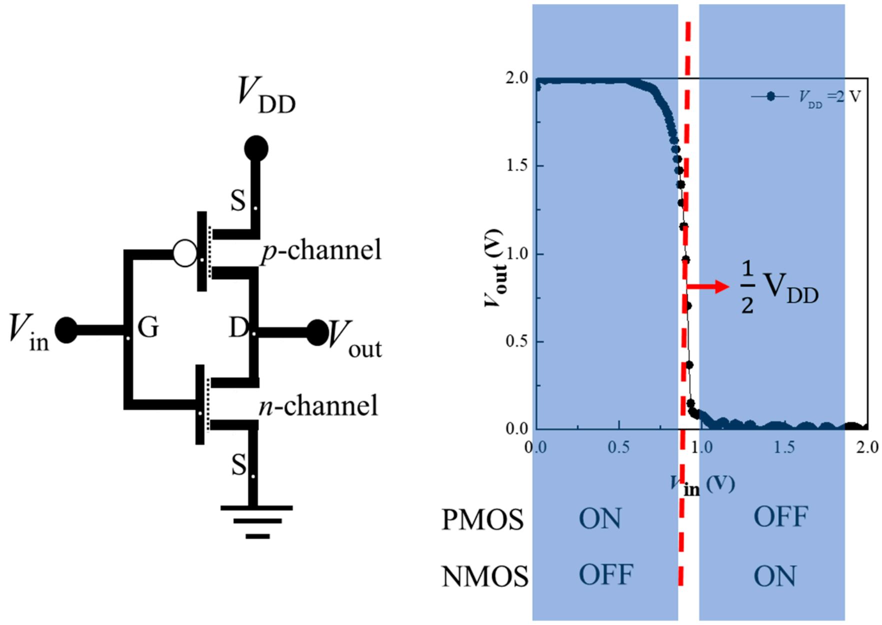

Figure S3. Schematic diagram of O-CMOS operation
